# Hyperuniformity and anti-hyperuniformity in one-dimensional substitution tilings

**DOI:** 10.1107/S2053273318015528

**Published:** 2019-01-01

**Authors:** Erdal C. Oğuz, Joshua E. S. Socolar, Paul J. Steinhardt, Salvatore Torquato

**Affiliations:** a School of Mechanical Engineering and The Sackler Center for Computational Molecular and Materials Science, Tel Aviv University, Tel Aviv, Israel; b Physics Department, Duke University, Durham, NC 27708, USA; c Department of Physics and Princeton Center for Theoretical Science, Princeton University, Princeton, NJ 08544, USA; d Center for Cosmology and Particle Physics, Department of Physics, New York University, New York, NY, 10003, USA; e Department of Chemistry, Department of Physics, Princeton Institute for the Science and Technology of Materials and Program in Applied and Computational Mathematics, Princeton University, Princeton, New Jersey 08540, USA

**Keywords:** substitution tiling, hyperuniformity, diffraction, limit-periodic tilings, non-Pisot tilings, quasiperiodic tilings

## Abstract

This work considers the scaling properties characterizing the hyperuniformity (or anti-hyperuniformity) of long-wavelength fluctuations in a broad class of one-dimensional substitution tilings. A simple argument is presented which predicts the exponent α governing the scaling of Fourier intensities at small wavenumbers, tilings with α > 0 being hyperuniform, and numerical computations confirm that the predictions are accurate for quasiperiodic tilings, tilings with singular continuous spectra and limit-periodic tilings. Quasiperiodic or singular continuous cases can be constructed with α arbitrarily close to any given value between −1 and 3. Limit-periodic tilings can be constructed with α between −1 and 1 or with Fourier intensities that approach zero faster than any power law.

## Abstract 2

This work examines the long-wavelength scaling properties of self-similar substitution tilings, placing them in their hyperuniformity classes. Quasiperiodic, non-PV (Pisot–Vijayaraghavan number) and limit-periodic examples are analyzed. Novel behavior is demonstrated for certain limit-periodic cases.

## 1. Introduction   

Recent work has shown that spatial structures with density fluctuations weaker at long wavelengths than those of a typical random point set may have desirable physical properties, and such structures are said to be *hyperuniform* (Torquato & Stillinger, 2003 ▸). Crystals and quasicrystals are hyperuniform, as are a variety of disordered systems, including certain equilibrium structures, products of nonequilibrium self-assembly protocols and fabricated metamaterials. [For examples, see Man *et al.* (2013 ▸), Haberko & Scheffold (2013 ▸), Dreyfus *et al.* (2015 ▸), Torquato *et al.* (2015 ▸), Hexner & Levine (2015 ▸), Castro-Lopez *et al.* (2017 ▸), Torquato (2018 ▸).] One approach to generating point sets with nontrivial spatial fluctuations is to use substitution tilings as templates. Our aim in this article is to characterize the degree of hyperuniformity in such systems and thereby provide design principles for creating hyperuniform (or anti-hyperuniform) point sets with desired scaling properties.

Substitution tilings are self-similar, space-filling tilings generated by repeated application of a rule that replaces each of a finite set of tile types with scaled copies of some or all of the tiles in the set (Frank, 2008 ▸). We are interested in the properties of point sets formed by decorating each tile of the same type in the same way. We consider here only one-dimensional (1D) tilings. Although generalization to higher dimensions would be of great interest, the 1D case already reveals important conceptual features.

Substitution rules are known to produce a variety of structures with qualitatively different types of structure factors 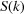. Some rules generate periodic or quasiperiodic tilings, in which case 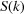 consists of Bragg peaks on a reciprocal-space lattice supported at sums and differences of a (small) set of basis wavevectors, which in the quasiperiodic case form a dense set. Others produce limit-periodic structures consisting of Bragg peaks located on a different type of dense set consisting of wavenumbers of the form 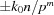, where *n*, *m* and *p* are positive integers (Godrèche, 1989 ▸; Baake & Grimm, 2011 ▸, 2013 ▸). Still others produce structures for which 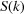 is singular at a dense set of points but does not consist of Bragg peaks (Bombieri & Taylor, 1986 ▸; Godrèche & Luck, 1992 ▸; Baake *et al.*, 2017 ▸). (A singular continuous spectrum has support on some set of zero Lebesgue measure, but has no finite weight at any single point.) We note in particular that a detailed analysis of the spectrum of substitution tilings with non-PV properties (defined below) reveals multifractal scaling laws (Godrèche & Luck, 1992 ▸). Finally, there are cases for which 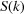 is absolutely continuous (Baake & Grimm, 2012 ▸) or the nature of the spectrum has not been clearly described.

In this article, we present a simple ansatz that predicts the scaling properties relevant for assessing the hyperuniformity (or anti-hyperuniformity) of 1D substitution tilings. We illustrate the validity of the ansatz via numerical computations for a variety of example tilings that fall in different classes with respect to hyperuniformity measures. We also delineate the full range of behaviors that can be obtained using the substitution construction method, including a novel class in which the integrated Fourier intensity 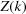 decays faster than any power as *k* approaches zero.

Section 2 reviews the definition of the scaling exponent α associated with both 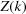 and the variance 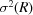 in the number of points covered by a randomly placed interval of length 2*R*. We then review the classification of tilings based on the value of α. Section 3 reviews the substitution method for creating tilings, using the well known Fibonacci tiling as an illustrative example. The substitution matrix 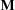 is defined and straightforward results for tile densities are derived. Section 4 presents a heuristic discussion of the link between density fluctuations in the tilings and the behaviors of 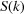 and 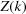, which leads to a prediction for α. The prediction is shown to be accurate for example tilings of three qualitatively distinct types (Torquato, 2018 ▸): strongly hyperuniform (class I), weakly hyperuniform (class III) and anti-hyperuniform. Section 5 shows, based on the heuristic theory, that the range of possible values of α produced by 1D substitution rules is 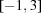 and that this interval is densely filled. Section 6 considers substitutions that produce limit-periodic tilings. Examples are presented of four distinct classes: logarithmic hyperuniform (class II), weakly hyperuniform (class III), anti-hyperuniform, and an anomalous class in which 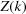 approaches zero faster than any power law. Finally, Section 7 provides a summary of the key results, including a table showing which types of tilings can exhibit the various classes of (anti-)hyperuniformity.

## 2. Classes of hyperuniformity   

For systems having a structure factor 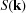 that is a smooth function of the wavenumber *k*, 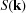 tends to zero as *k* tends to zero (Torquato & Stillinger, 2003 ▸), typically scaling as a power law: 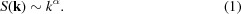
In 1D, a unified treatment of standard cases with smooth 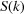 and quasicrystals with dense but discontinuous 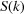 is obtained by defining α in terms of the scaling of the integrated Fourier intensity: 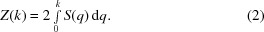
The factor of 2 is inserted for consistency with higher-dimensional generalizations where *q* is treated as a radial coordinate. In both cases, α may be defined by the relation (Oğuz *et al.*, 2017 ▸) 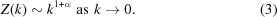
Systems with 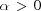 have long-wavelength spatial fluctuations that are suppressed compared with Poisson point sets and are said to be hyperuniform (Torquato & Stillinger, 2003 ▸). Prototypical strongly hyperuniform systems (with 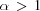) include crystals and quasicrystals. We refer to systems with 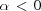 as anti-hyperuniform (Torquato, 2018 ▸). Prototypical examples of anti-hyperuniformity include systems at thermal critical points.

An alternate measure of hyperuniformity is based on the local number variance of particles within a spherical observation window of radius *R* (an interval of length 2*R* in the 1D case), denoted by 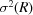. If 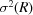 grows more slowly than the window volume (proportional to *R* in 1D) in the large-*R* limit, the system is hyperuniform. The scaling behavior of 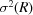 is closely related to the behavior of 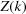 for small *k* (Torquato & Stillinger, 2003 ▸; Oğuz *et al.*, 2017 ▸). For a general point configuration in 1D with a well-defined average number density ρ, 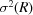 can be expressed in terms of 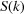 and the Fourier transform 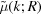 of a uniform density interval of length 2*R*: 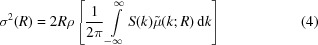
with 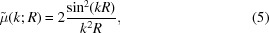
where ρ is the density. [See Torquato & Stillinger (2003 ▸) for the generalization to higher Euclidean space dimensions.] One can express the number variance alternatively in terms of the integrated intensity (Oğuz *et al.*, 2017 ▸): 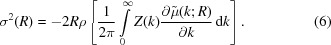



For any 1D system with a smooth or quasicrystalline structure factor, the scaling of 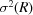 for large *R* is determined by α as follows (Torquato & Stillinger, 2003 ▸; Zachary & Torquato, 2009 ▸; Torquato, 2018 ▸): 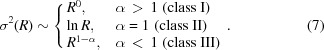
For hyperuniform systems, we have 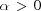, and the distinct behaviors of 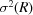 define the three classes, which we refer to as strongly hyperuniform (class I), logarithmic hyperuniform (class II) and weakly hyperuniform (class III). As mentioned above, systems with 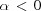 are classified as anti-hyperuniform.

The bounded number fluctuations of class I occur trivially for 1D periodic point sets (crystals) and are also known to occur for certain quasicrystals, including the canonical Fibonacci tiling described below (Oğuz *et al.*, 2017 ▸). Other quasiperiodic point sets (not obtainable by substitution) are known to belong to class II (Kesten, 1966 ▸; Aubry *et al.*, 1987 ▸; Oğuz *et al.*, 2017 ▸).

## 3. Substitution tilings and the substitution matrix   

A classic example of a substitution tiling is the 1D Fibonacci tiling composed of two intervals (tiles) of length *L* and *S*. The tiling is generated by the rule 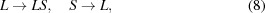
which leads to a quasiperiodic sequence of *L* and *S* intervals. An important construct for characterizing the properties of the tiling is the *substitution matrix*: 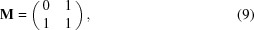
which acts on the column vector 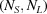 to give the numbers of *S* and *L* tiles resulting from the substitution operation.

**Figure 1:**
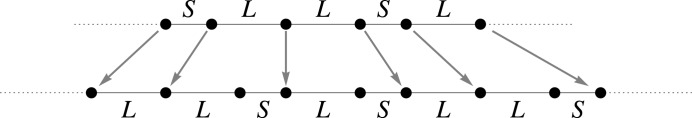
The Fibonacci substitution rule. The tiling on the upper line is uniformly stretched, then additional points are added to form tiles congruent to the originals.

If the lengths *L* and *S* are chosen such that the ratio 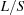 remains fixed, which in the present case requires 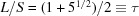, the substitution operation can be viewed as an affine stretching of the original tiling by a factor of τ followed by the division of each stretched *L* tile into an *LS* pair, as illustrated in Fig. 1 ▸. Given a finite sequence with 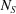 tiles of length *S* and 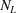 tiles of length *L*, the numbers of *L*’s and *S*’s in the system after one iteration of the substitution rule are given by the action of the substitution matrix on the column vector 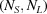.

More generally, substitution rules can be defined for systems with more than two tile types, leading to substitution matrices with dimension 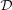 greater than 2. We present explicit reasoning here only for the 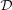 = 2 case. A substitution rule for two tile types is characterized by a substitution matrix: 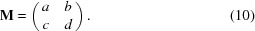
The associated rule may be the following: 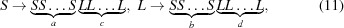
but different orderings of the tiles in the substituted strings are possible, and the choice can have dramatic effects. Note, for example, that the rule 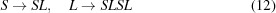
produces the periodic tiling 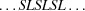, while the rule 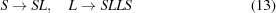
produces the more complicated sequence discussed below in Section 6.

Defining the substitution tiling requires assigning finite lengths to *S* and *L*. We let ξ denote the length ratio 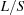, and we consider only cases where the substitution rule preserves this ratio [*i.e.* 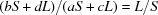] so that the rule can be realized by affine stretching followed by subdivision. This requires 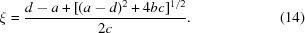
For all discussions and plots below, we measure lengths in units of the short tile length, *S*.

The *SL* sequence generated by a substitution rule is obtained by repeated application of that operation to some seed, which we will take to be a string containing 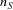 short intervals and 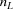 long ones. We are interested in point sets formed by decorating each *L* tile with 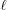 points and each *S* tile with *s* points. The total number of points at the *m*th iteration is 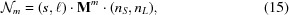
and the length of the tiling at the same step is 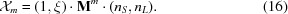
Let 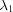 and 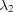 be the eigenvalues of 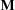, with 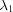 being the largest, and let 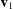 and 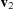 be the associated eigenvectors. We have
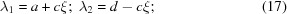


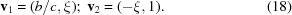
The unit vectors 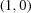 and 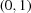 may be expressed as follows:
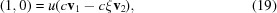


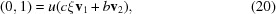
where 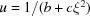. We then have 
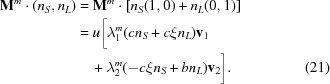
The density of tile vertices after *m* iterations, 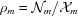, is thus
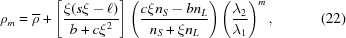
with 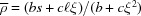, where we have used the fact that 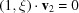.

## 4. Scaling properties of 1D substitution tilings   

As long as the coefficient of 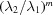 in equation (22) does not vanish, the deviations of ρ from 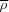 for portions of the tiling that are mapped into each other by substitution are related by 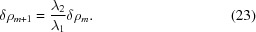
If the coefficient does vanish, which requires that ξ be rational, the tiling may be periodic, but the ordering of the intervals in the seed becomes important. We will revisit this point below. For now we assume that the tiling is not periodic.

We make three conjectures regarding nonperiodic substitution tilings, supported, as we shall see, by numerical experiments. The results are closely related to recently derived rigorous results (Baake, Gaehler *et al.*, 2018 ▸).

*Conjecture 1*. We take equation (23) to be the dominant behavior of density fluctuations throughout the system, not just for the special intervals that are directly related by substitution. That is, we assume that there exists a characteristic amplitude of the density fluctuations at a given length scale after averaging over all intervals of that length, and that the 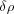 in equation (23) can be interpreted as that characteristic amplitude.

*Conjecture 2*. For the present purposes, we define the real-space density 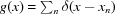 to consist of unit-strength δ-functions placed at every point 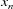 where two tiles meet, and consider the Fourier amplitudes 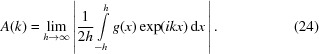
With this definition, the average density over a large domain is equal to the number density of tiles, ρ, in that domain. We assume that 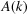 scales the same way as the density fluctuations at the corresponding length scale: 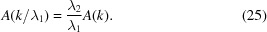 This implies the form 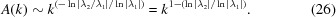
Squaring to get 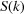, we have 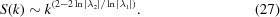
This conjecture may not hold when interference effects are important, as in the case discussed in Section 6 below.

*Conjecture 3*. While 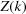 is an integral of 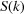, the exponent must be calculated carefully when 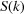 consists of singular peaks. In the Fibonacci projection cases, the scaling of peak positions and intensities conspires to make 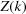 scale with the same exponent as the envelope of 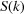 (Oğuz *et al.*, 2017 ▸). We assume that this property carries over to substitution tilings with more than one eigenvalue greater than unity. Though the diffraction pattern is not made up of Bragg peaks (Bombieri & Taylor, 1986 ▸; Godreche & Luck, 1990 ▸), we conjecture that it remains sufficiently singular for the relation to hold. Thus we immediately obtain 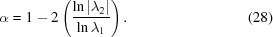
Note that this calculation of the scaling exponent makes no reference to the distinction between substitutions with 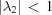 and those with 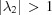. In the former case, 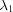 is a Pisot–Vijayaraghavan (PV) number, 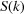 consists of Bragg peaks and 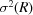 remains bounded for all *R*. In the latter case, the form of 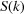 is more complex (Bombieri & Taylor, 1986 ▸), and quantities closely related to 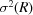, including the ‘wandering exponent’ associated with lifts of the sequence onto a higher-dimensional hypercubic lattice, are known to show nontrivial scaling exponents (Godreche & Luck, 1990 ▸).

From equation (28), we see that the hyperuniformity condition 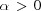 requires 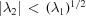. Though the result was obtained for substitutions with only 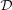 = 2 tile types, it holds for 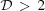 as well, so long as all ratios of tile lengths are preserved by the substitution rules; *i.e.* the dominant contribution to the long-wavelength fluctuations still scales like 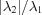. This distinction between hyperuniform and anti-hyperuniform substitution tilings thus divides the non-PV numbers into two classes which, to our knowledge, have not previously been identified as significantly different. We note, for example, that the analysis presented in Baake, Grimm *et al.* (2018 ▸), which treats substitution matrices of the form 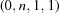 and shows that they have singular continuous spectra (having no Bragg component or absolutely continuous component) for 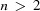, does not detect any qualitative difference between the cases 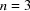 and 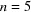. The former case is hyperuniform, with 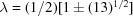 and 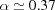, while the latter is anti-hyperuniform, with 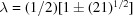 and 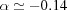.

**Figure 2:**
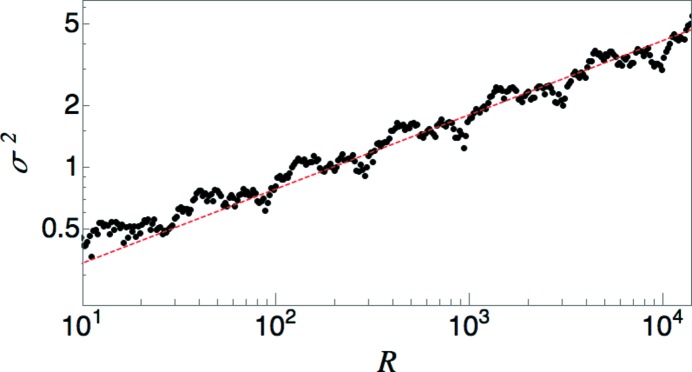
Log–log plot of the number variance (black dots) for a non-PV substitution tiling corresponding to 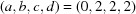 decorated with points of equal weight at each tile boundary. The variance was computed numerically for the tiling created by 12 iterations of the substitution on the initial seed *SL*. The red dashed line has the predicted slope 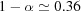.

For the Fibonacci case, we have 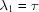 and 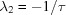, yielding 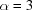, which agrees with the explicit calculation in Oğuz *et al.* (2017 ▸). Considering 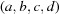 of the form 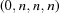 for arbitrary *n*, we find cases that allow explicit checks of our predictions for α for both hyperuniform and anti-hyperuniform systems. We have 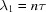 and 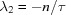, yielding
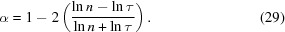
For 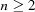, the presence of more than one eigenvalue with magnitude greater than unity gives rise to more complex spectral features, possibly including a singular continuous component. For 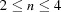, our calculation predicts 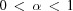 and hence 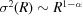. We numerically verify the latter result for 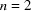 using a set of 954 369 points generated by 12 iterations of the substitution tiling, where the decoration consists of placing one point at the rightmost edge of each tile (with 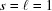). Fig. 2 ▸ shows the computed number variance. For each point, a window of length 2*R* is moved continuously along the sequence and averages are computed by weighting the number of points in the window by the interval length over which that number does not change. A regression analysis yields 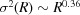, in close agreement with the predicted exponent from equation (27): 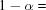 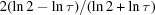 ≃ 0.36094.

**Figure 3:**
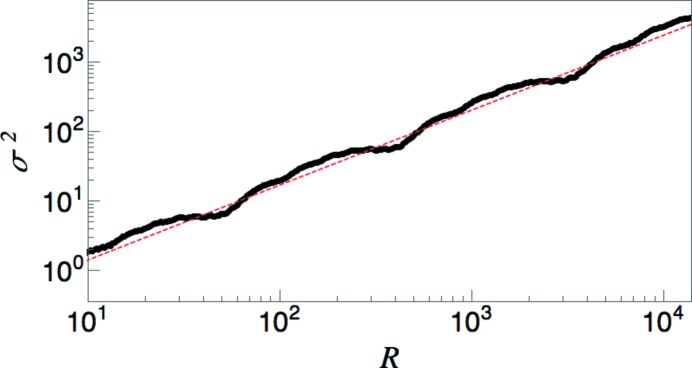
Log–log plot of the number variance (black dots) for an anti-hyperuniform substitution tiling corresponding to 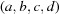 = 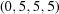 decorated with points of equal weight at each tile boundary. The variance was computed numerically for the tiling created by six iterations of the substitution on the initial seed *SL*. The red dashed line has the predicted slope 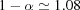.

For 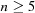, the calculated value of α is negative, approaching 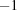 as *n* approaches infinity. The point set is therefore anti-hyperuniform; it contains density fluctuations at long wavelengths that are stronger than those of a Poisson point set. For 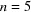, we have 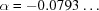. Fig. 3 ▸ shows a log–log plot of the computed number variance along with the line corresponding to 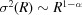. Again, the agreement between the numerical result and the predicted value is quite good. Intuition derived from theories based on nonsingular forms of 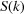 suggests that a negative value of α should be associated with a divergence in 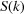 for small *k*, though it remains true that 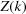 converges to zero for 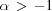. For singular spectra, the envelope of 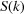 scales like 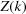, and we do not expect any dramatic change in the behavior of 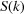 as α crosses from positive (hyperuniform) to negative (anti-hyperuniform). The theories presented in Baake *et al.* (2017 ▸) and Godreche & Luck (1990 ▸) may provide a path to the computation of scaling properties of 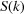 in these cases. It is worth noting, however, that the various classes of behavior can be realized by substitutions that produce limit-periodic tilings with 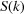 consisting entirely of Bragg peaks with no singular-continuous component, as shown in Section 6 below.

For rules that yield rational values of the length ratio ξ, the coefficient of 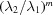 in equation (22) can vanish for appropriate choices of 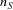 and 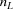, suggesting that there are no fluctuations about the average density that scale with wavelength. This reflects the fact that the sequence of intervals associated with the substitutions can be chosen to generate a periodic pattern. (A simple example is 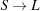 and 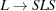, which generates the periodic sequence 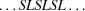, with 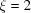, 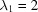 and 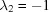.) For such cases, 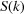 is identically 0 for all *k* smaller than the reciprocal-lattice basis vector. For other interval sequence choices corresponding to the same 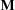, the tiling can be limit-periodic, and we would expect the scaling to be given by applying the above considerations with generic choices of the ordering, which would yield 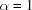 and therefore a logarithmic scaling of 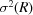. This case is presented in more detail in Section 6 below, and the logarithmic scaling is confirmed.

## 5. Achievable values of α   

Beyond establishing that substitution tilings exist for each hyperuniformity class, it is natural to ask whether any desired value of α can be realized by this construction method. Here we show that if 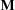 is full rank, α always lies between 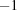 and 3.

First, note that the maximum value of 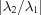 is 1, by definition, which sets the lower bound on α via equation (28). The upper bound on α is obtained when 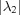 is as small as possible, but there is a limit on how small this can be. The product of the eigenvalues of 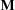 is equal to 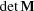, so 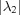 cannot be smaller than 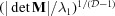. But 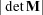 is an integer, and the smallest nonzero value it can take is 1. (The case 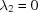 is discussed in Section 6 below. For 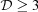, one can have 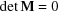 with nonzero 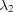. The analysis of such cases is beyond our present scope.) Hence we have 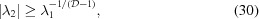
implying 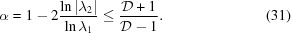
Thus the maximum value of α obtainable by this construction method is 3, which can occur for 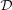 = 2, as in the Fibonacci case.

The family of substitutions considered in Section 4 above produces a discrete set of values of α ranging from 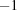 to 3. By considering two additional families, we can show that the possible values of α densely fill this interval. For 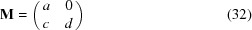
with 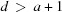 and 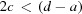, we have 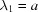 and 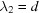. Note that 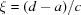 is rational here; we assume that the substitution sequences for the two tiles are chosen so as to avoid periodicity. We have 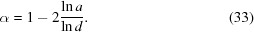
For fixed *a*, *d* can range from 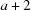 to 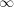. As *d* approaches infinity, α approaches 1. For 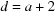, as *a* approaches infinity, α approaches 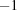. For sufficiently large *d*, the values of *a* between 1 and 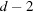 yield an arbitrarily dense set of α’s between 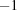 and 1.

Another class of 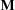
’s produces α’s between 1 and 3. For 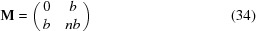
with 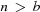, we have
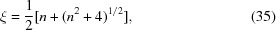


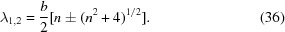
We thus obtain 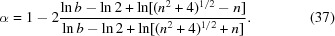
For large *n*, we have 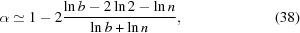
which approaches 3 for 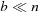 and approaches 1 for 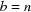. By making *n* as large as desired, the values of *b* between 1 and *n* give α’s that fill the interval between 1 and 3 with arbitrarily high density.

## 6. Limit-periodic tilings   

For a limit-periodic tiling, the set of tiles is a union of periodic patterns with ever-increasing lattice constants of the form 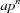, where *p* is an integer and *n* runs over all positive definite integers (Godrèche, 1989 ▸; Baake & Grimm, 2011 ▸, 2013 ▸; Socolar & Taylor, 2011 ▸). We show here that there exist limit-periodic tilings of four hyperuniformity classes: logarithmic (class II), weakly hyperuniform (class III), anti-hyperuniform, and an anomalous case in which 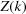 decays to zero faster than any power law as *k* goes to zero. The latter corresponds to a rule for which 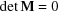 (and 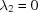), in which case α is not well defined. The existence of anti-hyperuniform limit-periodic tilings shows that anti-hyperuniformity does not require exotic singularities in 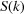 for small *k*. Generally, it requires only that 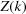 grows sub-linearly with *k*.

### 6.1. The logarithmic case (α = 1)   

**Figure 4:**
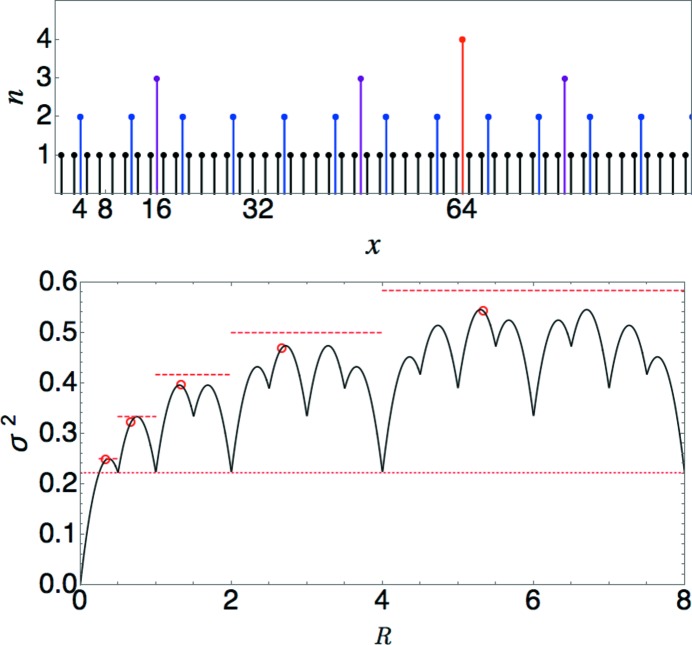
The 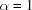 (period doubling) limit-periodic tiling. Top: the tile boundaries with each point plotted at a height corresponding to the value of *n* for the sublattice to which it belongs. Bottom: plot of the number variance. The horizontal dotted line marks 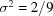, which is obtained for every *R* of the form 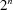 with integer 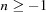. The dashed lines indicate upper bounds, and the open circles are analytically calculated values for 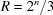. See text for details.

The rule 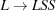, 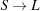 with 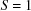 and 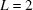 yields the well-known ‘period doubling’ limit-periodic tiling. The eigenvalues of the substitution matrix are 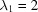 and 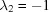, leading to the prediction 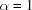 and therefore quadratic scaling of 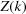 and logarithmic scaling of 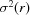. Numerical results for 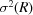 are in good agreement with this prediction (Torquato *et al.*, 2018 ▸). In fact, one can show explicitly via direct calculation of 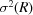 that the scaling is logarithmic. The calculation outlined in Appendix *A* [App appa] shows that 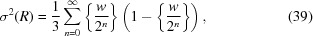
where 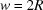 and 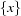 denotes the fractional part of *x*. From this it follows that for 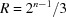 with 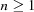 we have 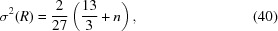
demonstrating clear logarithmic growth for this special sequence of *R* values. One can also derive an upper bound over the interval 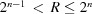 by assuming that the summand in equation (39) takes its maximum possible value on the intervals 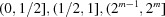, for 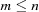, and maximizing the possible sum of the exponentially decaying remaining contributions. The result is 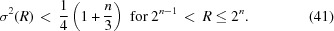
This upper bound also grows logarithmically and is shown as a series of dashed lines in Fig. 4 ▸.

**Figure 5:**
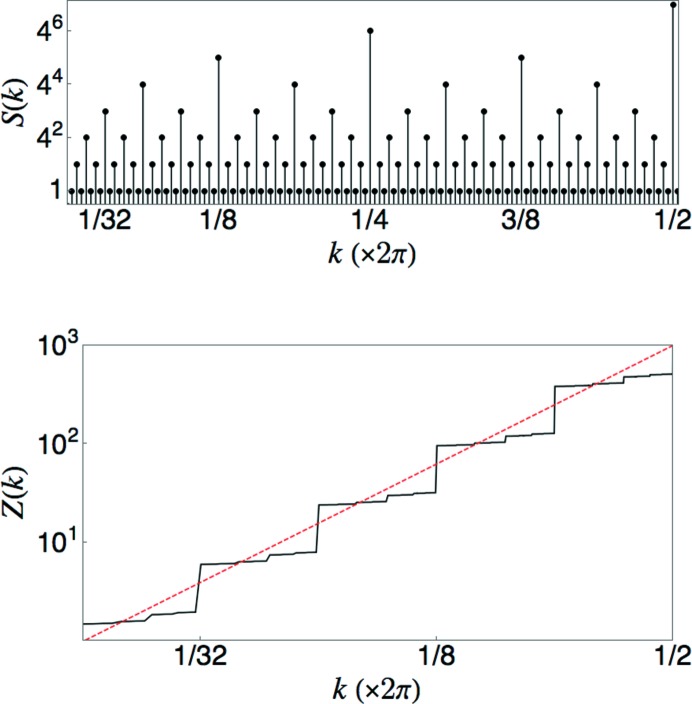
The 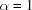 limit-periodic tiling. Top: a logarithmic plot of the analytically computed 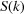 (arbitrarily scaled) including 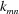 with 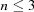. Bottom: a log–log plot of 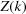 computed numerically from 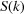. The dashed red line shows the expected quadratic scaling law.

It is instructive to carry out a more detailed analysis of 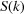 for this particularly simple case as well. [See also Torquato *et al.* (2018 ▸).] The tiling generated by applying the substitution rule repeatedly to a single *L* with its left edge at 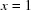 consists of points located at positions 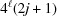, where 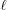 and *j* range over all positive integers (including zero). The structure factor therefore consists of peaks at 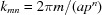, with 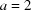 and 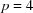, for arbitrarily large *n* and all integer *m*. For *m* not a multiple of 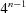, the peak at 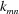 gets nonzero contributions only from the lattices with 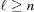. These can be summed as follows: 
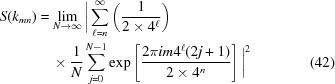


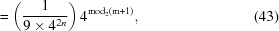
where the factor of 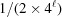 in the first line is the density of the sublattice with that lattice constant. Applying this reasoning to each value of *n* gives a result that can be compactly expressed as 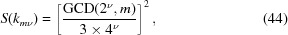
where ν is an arbitrarily large integer, 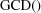 is the greatest common denominator function, and *m* can now take any positive integer value. Fig. 5 ▸ shows plots of 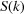 and 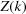 for this tiling. [See also Torquato *et al.* (2018 ▸) for an explicit expression for 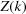 and proof of the quadratic scaling.] Note that the apparent repeating unit in the plot of 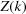 spans only a factor of 2, even though the scaling factor for the lattice constants is 4. A similar effect occurs in the Poisson and anti-hyperuniform cases below. In the present case, the construction in Appendix *A* [App appa] showing that the density can be expressed using lattice constants 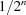 explains the origin of the effect.

### 6.2. A Poisson scaling example (α = 0) and weak hyper­uniformity (0 < α < 1)   

**Figure 6:**
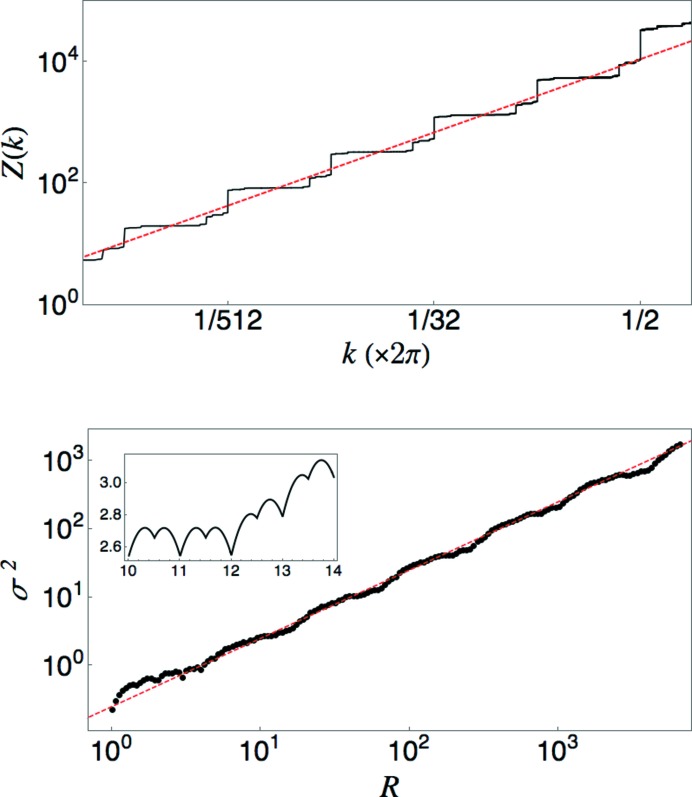
Comparison of direct computation of 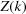 and 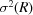 with the predicted scaling laws for a limit-periodic tiling with 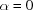. The dashed red lines show the expected linear scaling laws. The inset shows the piecewise parabolic behavior of 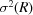 over a small span of *R* values.

The substitution rule 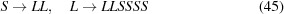
with 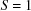 and 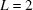 produces a limit-periodic tiling with 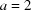 and 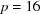. Equation (28) yields 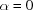, which is the value corresponding to a Poisson system. Fig. 6 ▸ shows the result of direct computations of 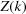 including all of the Bragg peaks at 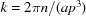 and of 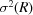. Values of 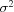 were computed from a sequence of 21 889 points obtained by seven iterations of the substitution rule on an initial *L* tile. For each point, a window of length 2*R* is moved continuously along the sequence for the computation of the averages.

Limit-periodic examples of weak hyperuniformity (class III) are afforded by substitutions of the form 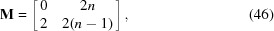
with 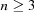 with 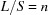, which yields 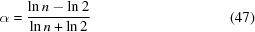


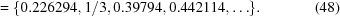



### 6.3. Anti-hyperuniformity (α < 0)   

**Figure 7:**
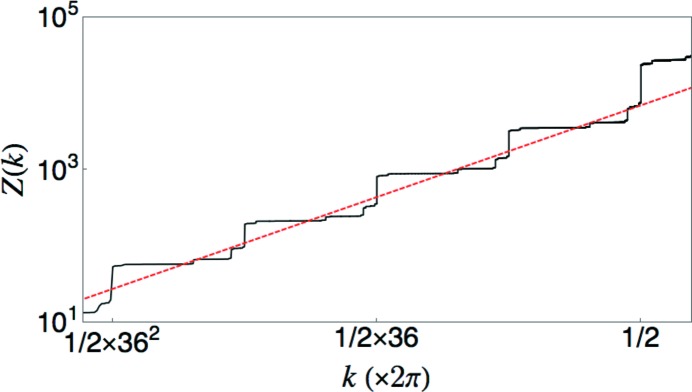
Comparison of direct computation of 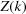 with the predicted scaling law for a limit-periodic tiling with 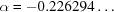. The dashed red line shows the expected scaling law with slope 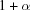.

The substitution rule 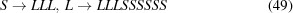
with 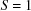 and 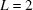 produces a limit-periodic tiling with 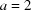 and 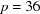. Equation (28) yields 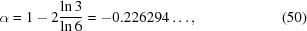
which indicates anti-hyperuniform fluctuations. Fig. 7 ▸ shows the result of a direct computation of 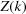 including all of the Bragg peaks at 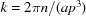.

More generally, substitution matrices of the form 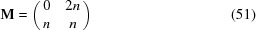
with 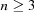 and 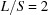 yield limit-periodic anti-hyperuniform tilings with 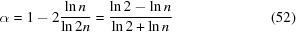


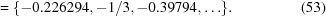



### 6.4. A λ_2_ = 0 case (α undefined)   

A special class of tilings is derived from substitution matrices of dimension 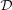 = 2 that have 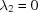 (and hence 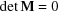). Such rules can produce periodic tilings, limit-periodic ones or more complex structures. The criteria for limit-periodicity can be obtained by analyzing constant-length substitution rules in which each *L* is considered to be made up of two tiles of unit length: 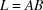. If the induced substitution rule on *S*, *A* and *B* exhibits appropriate coincidences, the tiling is limit-periodic (Dekking, 1978 ▸; Queffelec, 1995 ▸). For the substitution matrix 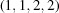, the rule 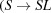; 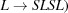 produces a periodic tiling, and 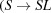; 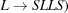, for example, produces a limit-periodic tiling.

For the limit-periodic cases, the analysis above would suggest 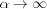, or, more properly, α is not well defined. We present here an analysis of a particular case for which the convergence of 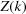 to zero is indeed observed to be faster than any power law.

**Figure 8:**
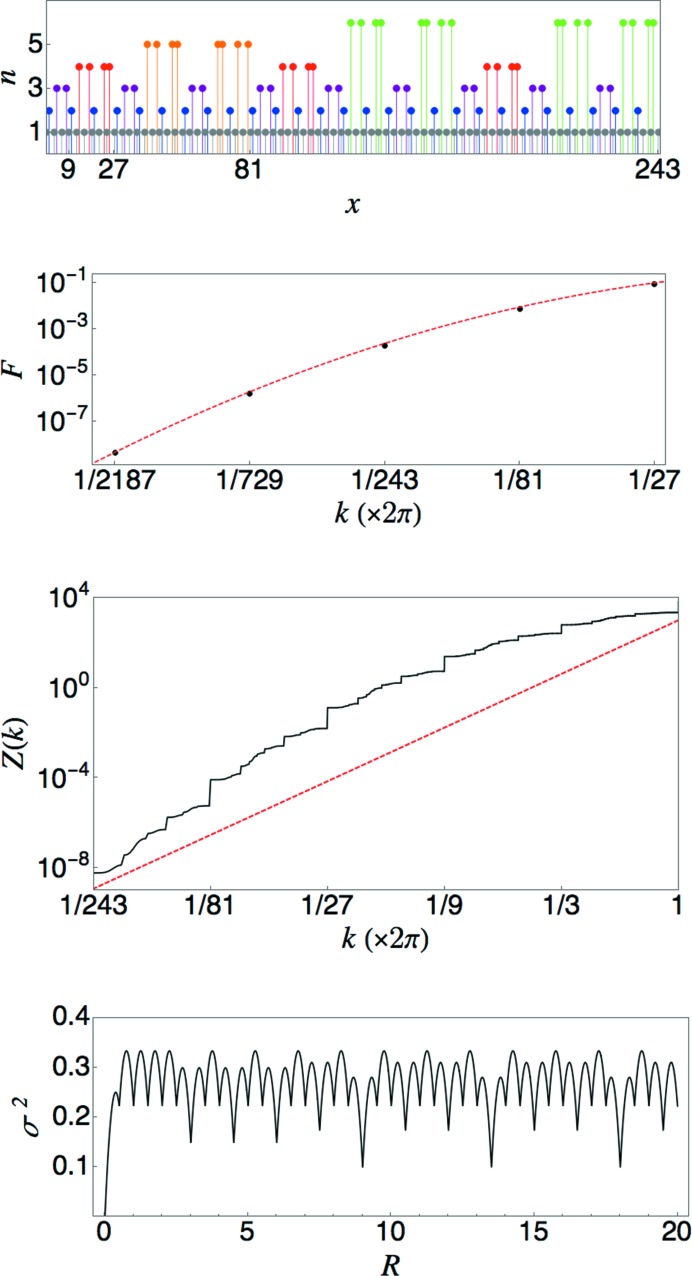
Top: periodic sublattices of the limit-periodic point set generated by equation (54). Each point is plotted at a height *n* corresponding to the subset that contains it. Points of the same color form a periodic pattern with period 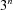. Second: deviation of 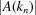 from 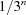. Third: the integrated structure factor for the limit-periodic tiling with 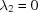, computed from subsets with 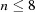. The straight red (dashed) line of slope 5 is a guide to the eye for observing the concavity of the curve. Bottom: plot of the number variance for the limit-periodic tiling with 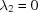.

The substitution rule 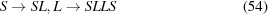
with 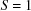 and 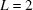 produces a limit-periodic tiling with 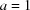 and 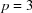. Inspection of the point set (displayed in Fig. 8 ▸) reveals that the number of points in the basis of each periodic subset for 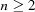 is 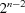. The density of points in subset 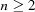 is 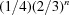. The substitution matrix 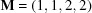 has eigenvalues 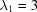 and 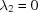.

The unusual scaling in this case arises from interference effects associated with the form factors of the different periodic subsets. Let 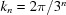, the fundamental wavenumber for the *n*th subset, and let 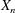 denote the set of points in a single unit cell of the *n*th subset. 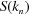 has contributions coming from all subsets of order *n* and higher. (Subsets of lower order do not contribute, as their fundamental wavenumber is larger than 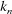.) After some algebra, we find
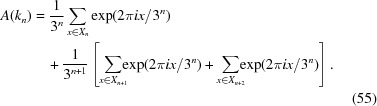
Numerical evaluation of the sums over the unit-cell bases reveals that 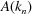 is suppressed by the interference from subsets of higher order. Fig. 8 ▸ shows the behavior of the quantity 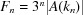, revealing a rapid decay for small 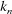. The red (dashed) line shows the curve 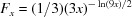, which appears to fit the points well. An analytic calculation of 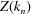 is beyond our present reach. The middle panel of Fig. 8 ▸ shows the results of a numerical computation that includes all peaks 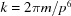, with 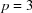. It is clear that 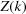 is concave downwards on the log–log plot, consistent with the expectation that 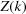 goes to zero faster than any power of *k*. Note that the curve is not reliable for the smallest values of *k* due to the cutoff on the resolution of *k* values that are included. The deviation from power-law scaling is most easily seen in the increasing with *n* of the step sizes of the large jumps at 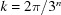. (Compare with the constant step sizes in Figs. 5 ▸, 6 ▸ and 7 ▸.)

For completeness, the bottom panel of Fig. 8 ▸ also shows a plot of the number variance for this tiling. As expected, 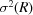 is bounded from above. We note that the curve appears to be piecewise parabolic, which is also the case for the standard Fibonacci quasicrystal (Oğuz *et al.*, 2017 ▸), though the technique for calculating 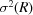 based on projecting the tiling vertices from a 2D lattice is not applicable here.

## 7. Discussion   

We have presented a heuristic method for calculating the hyperuniformity exponent α characterizing point sets generated by substitution rules that preserve the length ratios of the intervals between points. The calculation relies only on the relevant substitution matrix and an assumption that the tile order under substitution does not lead to a periodic tiling. The method performs well in that it yields a value of α consistent with direct measurements of the scaling of 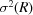 in several representative cases. This allows for a straightforward construction of point sets with any value of α between 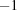 and 3.

It is well known that substitution rules can be divided into distinct classes corresponding to substitution matrices with eigenvalues that are not PV numbers leading to structure factors 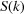 that are singular continuous (Bombieri & Taylor, 1986 ▸; Baake *et al.*, 2017 ▸), while substitution rules for which 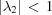 yield Bragg peaks. Our analysis shows that this distinction corresponds to α greater than or less than unity, respectively. From the perspective of hyperuniformity, on the other hand, the critical value of α is zero, which corresponds to 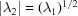. To achieve 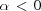, a naïve comparison to scaling theories for systems with continuous spectra would suggest that 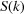 must diverge for small *k*. We find, however, that anti-hyperuniformity, which does require sub-linear scaling of 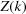, can occur without any divergence both in cases where the spectrum is singular continuous, as for non-PV substitutions, and in cases where the spectrum consists of a dense set of Bragg peaks, as in some limit-periodic systems.

Finally, our investigations led us to consider the results of applying substitution rules for which 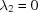, which turned up a novel case of a limit-periodic tiling for which 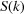 approaches zero faster than any power law. The physical implications of this type of scaling have yet to be explored.

The different tiling types and their hyperuniformity properties are summarized in Table 1 ▸. Examples of quasiperiodic tilings in classes I and II are presented in Oğuz *et al.* (2017 ▸). Note, however, that the class II case is not a substitution tiling. We do not know whether some other construction methods might yield quasiperiodic tilings that are in class III, anti-hyperuniform, or even anomalous. For non-PV tilings (which are substitution tilings by definition), at least two eigenvalues of the substitution matrix must be greater than unity, which rules out class II and class I. We conjecture that there are no limit-periodic tilings in class I. We can prove this for 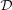 = 2 substitutions based on the fact that limit-periodicity requires the two eigenvalues to be rational and the fact that 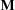 has only integer elements requires their sum and product to be integers, but we do not have a proof for 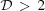.

## Figures and Tables

**Figure 9:**
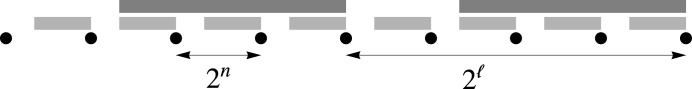
Illustration for explaining the computation of a term in the double sum expression for 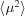.

**Table 1:** Types of 1D tilings and their possible hyperuniformity classes A tick indicates that tilings of the given type exist, a dash that there are no such tilings, and a question mark that we are not sure whether such tilings exist.

	Anti-hyperuniform	Weakly hyperuniform (class III)	Logarithmically hyperuniform (class II)	Strongly hyperuniform		
				Class I	Anomalous	Gapped
	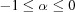	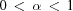	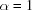	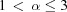	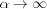	α irrelevant
Periodic	–	–	–	–	–	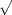
Quasiperiodic	?	?	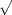	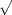	?	–
Non-PV	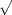	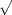	–	–	–	–
Limit-periodic	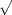	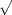	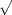	?	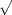	–

## Appendix A Calculation of σ^2^(*R*) for the period doubling limit-periodic tiling

The substitution rule ,  (with  and ) applied to an initial *L* with its left boundary at  produces a tiling with tile boundaries at all positions of the form , with *j* and *m* both running over all positive integers (including zero). We are interested in computing  for the density

Recall that  is the variance in the number of points covered by a window of length 2*R* placed with its left edge at *x* with uniform probability over all positive real values of *x*.

In Torquato *et al.* (2018 ▸), an expression for  is derived using equation (4) above. Here we show how  can be computed directly, thereby confirming the validity of equation (4) for this limit-periodic system and arriving at a particularly simple expression that can be analyzed in detail.

We first note that we can rewrite  as follows:

To see this, first note that if *x* is an odd integer, then the only term that contributes is , , which gives a . This is the  lattice of equation (56). More generally, if *x* is an odd multiple of , there are contributions only from all , and these have alternating signs. If *p* is odd, the number of such contributions is even, yielding a density of zero. If *p* is even, the sum of the contributions is . The even values of *p* correspond to all integer values of *m* in equation (56).

Let  be the length of the window and let  be the number of points in the *n*th lattice covered by the window. It is convenient to take the window to be open at its left edge and closed at its right edge. Define  as the fractional part of . It is convenient to think of *w* as being expressed in base 2.  is then given by the first *n* digits to the left of the decimal point, plus all of the digits to the right. Note that  depends on *x* only through ; the integer part of  adds the same number of points independent of the value of *x*. Furthermore, there are only two possible values of , which differ by unity. For the purpose of computing the variance, we take these to be 0 and 1, and we work with the densities of these values rather than the full values of .

If the window is placed with its left edge at , the contribution to the density from the *n*th lattice is 0. In order for the window to cover an additional point, the left edge must be placed such that . The average density covered by the window of length *w* is thus

The density squared is

A nonzero contribution to  arises from an individual term if and only if both the *n* and  lattices contribute. For , the fraction of *x*’s for which this is true is

The first term accounts for window placements that give a contribution from the *n*th lattice. The light-gray bars in Fig. 9 ▸ show the values of *x* where the left edge of the window can be placed to produce a nonzero contribution. The term in parentheses counts the number of such intervals that occur within a region that contribute from the th lattice (indicated by dark-gray bars in the figure) divided by the number of bars in one lattice spacing of the th lattice. We thus have

Using  and writing out  as a sum over *n* plus a double sum with , straightforward algebra with convenient cancelations of some of the double sums yields

This result has been confirmed to be in perfect agreement with direct computations.

Equation (64) describes a piecewise quadratic function of *w* (see Fig. 5 ▸). One immediately sees that all values of *w* of the form  give the same result; they give  for  and the same infinite series for . Recalling that , the shared value is

To show that the upper envelope of  grows logarithmically, we first prove an upper bound that grows only logarithmically, then identify a special sequence of window length values for which the growth is logarithmic. The upper bound is obtained by replacing all terms  in the sum with the maximum value  for all , then replacing the remaining infinite series with its maximal value, obtained by maximizing . The result is

To show that there is a sequence of *R* values for which  grows logarithmically, consider *w* of the form . Note that the binary representation of *w* is  for *n* odd and  for *n* even. Again we consider the contributions from , then sum the remaining series. The value of  oscillates between 1/3 and 2/3 for . Straightforward algebra yields

which clearly grows logarithmically with *R*. Note that the coefficient of *n* here is 2/27, reasonably close to the coefficient of 1/12 derived for the upper bound, and that, as shown in Fig. 5 ▸, these points are quite close to the true maxima for .
